# Therapeutic opportunities for pain medicines via targeting of specific translation signaling mechanisms

**DOI:** 10.1016/j.ynpai.2018.02.001

**Published:** 2018-02-23

**Authors:** Salim Megat, Theodore J. Price

**Affiliations:** School of Behavioral and Brain Sciences, The University of Texas at Dallas, USA

**Keywords:** mTOR, MNK, eIF4E, eIF4A, AMPK, Nociceptor, Sensitization

## Abstract

As the population of the world ages and as more and more people survive diseases that used to be primary causes of mortality, the incidence of severe chronic pain in most of the world has risen dramatically. This type of pain is very difficult to treat and the opioid overdose epidemic that has become a leading cause of death in the United States and other parts of the world highlights the urgent need to develop new pain therapeutics. A common underlying cause of severe chronic pain is a phenotypic change in pain-sensing neurons in the peripheral nervous system called nociceptors. These neurons play a vital role in detecting potentially injurious stimuli, but when these neurons start to detect very low levels of inflammatory meditators or become spontaneously active, they send spurious pain signals to the brain that are significant drivers of chronic pain. An important question is what drives this phenotypic shift in nociceptors from quiescence under most conditions to sensitization to a broad variety of stimuli and spontaneous activity. The goal of this review is to discuss the critical role that specific translation regulation signaling pathways play in controlling gene expression changes that drive nociceptor sensitization and may underlie the development of spontaneous activity. The focus will be on advances in technologies that allow for identification of such targets and on developments in pharmacology around translation regulation signaling that may yield new pain therapeutics. A key advantage of pharmacological manipulation of these signaling events is that they may reverse phenotypic shifts in nociceptors that drive chronic pain thereby creating the first generation of disease modifying drugs for chronic pain.

## Phenotypic changes in nociceptors drive chronic pain and require changes in gene expression

A key feature of many chronic pain states is a persistent change in the sensitivity of nociceptors that outlives the tissue healing process (Price and Inyang, 2015; Price and Gold, 2017). This results in hypersensitivity to mechanical stimulation, thermal hyperalgesia and, in many cases, spontaneous pain (Campbell and Meyer, 2006). Clinical studies consistently demonstrate that blocking peripheral input from sensitized nociceptors rapidly attenuates pain in chronic pain patients (Haroutounian et al., 2014; Vaso et al., 2014). These findings provide compelling evidence for the hypothesis that chronic pain requires a peripheral driver and that peripheral driver is likely to be sensitized or spontaneously active nociceptors. Preclinical studies also provide compelling evidence for this hypothesis. For instance, after a nerve injury that causes neuropathic pain, axons that sprout back into the injured area are sensitized to mechanical stimulation and this sensitization persists even after these nerve endings re-innervate their target organ (Jankowski et al., 2009). Very recent evidence suggests that mechanically gated channels in A-type nociceptors change their gating properties after injury providing a possible biophysical basis for some forms of mechanical hypersensitivity after injury (Weyer et al., 2015). Mechanisms governing thermal hyperalgesia are now very well understood and involve alterations in the function of TRPV1 (for heat) and TRPM8 (for cold). Finally, the biophysical basis of injury-induced emergence of spontaneous activity in nociceptors is starting to be elucidated (Price and Gold, 2017). This involves changes in expression (Tsantoulas et al., 2012; Laumet et al., 2015; Calvo et al., 2016) and function of voltage-gated channels (Gold et al., 2003) that leads to subthreshold membrane oscillations which subvert the stability of the resting membrane potential making these neurons susceptible to ectopic action potential generation (Devor, 2006).

**Fig. 1:**
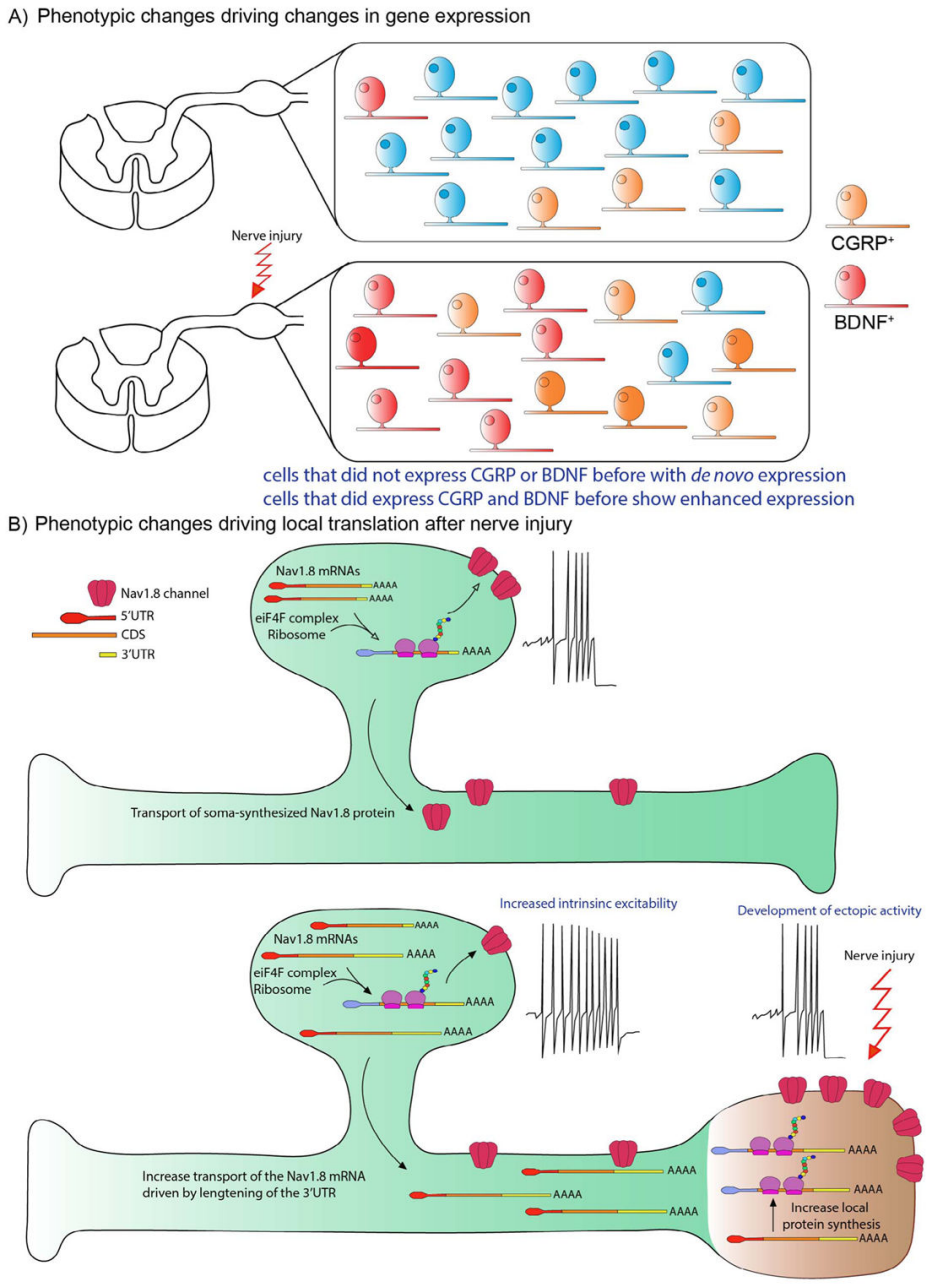
Phenotypic changes in DRG neurons associated with nerve injury and neuropathic pain A) Nerve injury can produce phenotypic changes leading to changes in expression for a variety of different peptides or proteins, including BDNF or CGRP. These include changes in expression in cells that already expressed these genes (brighter colors) or *de novo* expression in cells that did not previously express these genes. B) A second sort of phenotypic change involves altered translational control. For instance, after nerve injury Nav1.8 mRNA is increasingly trafficked into the axon and is locally translated at sites of injury contributing to altered excitability and potentially ectopic discharges.

Key questions emerging from these findings are: how do these injury-induced changes in nociceptor function occur and why do they so frequently persist after an injury has healed. The central theses of this review are: 1) that these changes in nociceptor function require changes in gene expression, 2) that these changes in gene expression can be persistent once they are turned on, 3) that these changes in gene expression are regulated at the level of transcription and at the level of translation and 4) that transcriptional and translational regulation are likely to be uncoupled, creating a unique opportunity to pharmacologically target translational regulation to reverse chronic pain states. Phenotypic changes in gene expression in nociceptors have been widely studied since the advent of the use of molecular biology techniques in pain neurobiology. Some of the earliest evidence of phenotypic changes in nociceptors after injury involved altered expression of neuropeptides (Fig. 1A) like calcitonin gene-related peptide (CGPR, (McMahon et al., 1995; Seybold et al., 1995; Ma and Quirion, 2006)) and brain-derived neurotrophic factor (BDNF, (Mannion et al., 1999)). An interesting feature of these changes is that peptide expression is increased in populations of neurons that already expressed these peptides and expression also appears *de novo* in cells that did not previously express these peptides. More recent work has focused on expression of voltage gated sodium and potassium channels (VGNaCs and VGKCs, respectively) in the context of neuropathic pain. Phenotypic changes in expression of VGNaCs have been observed for Nav1.3 and Nav1.6 and these usually involve a gain of function in expression (Waxman and Zamponi, 2014). On the other hand, a number of VGKCs are down-regulated after injury through mechanisms that involve epigenetic regulation of transcription for mRNAs that encode these channels (Tsantoulas et al., 2012; Laumet et al., 2015). The net results of these phenotypic shifts in VGC expression are an increase in VGNaCs and a decrease in VGKCs, which may be a causative factor in subthreshold membrane oscillations that cause ectopic action potential firing (Devor, 2006; Waxman and Zamponi, 2014; Price and Gold, 2017).

## Evidence that translation regulation signaling is critical for changes in nociceptor excitability

While altered transcription of VGCs, in particular VGKCs, is a compelling explanation for the emergence and persistence of chronic pain, other findings require more nuanced explanations. An excellent example is the role of Nav1.8 in neuropathic pain. While knockout mice for this VGNaC show normal mechanical hypersensitivity after nerve injury (Akopian et al., 1999), there is a clear redistribution of Nav1.8 protein to lesion sites (Gold et al., 2003), including neuromas, and ectopic and evoked activity from neuromas requires Nav1.8 expression (Roza et al., 2003). Moreover, in wild-type (WT) animals, knockdown of Nav1.8 expression or pharmacological blockade of Nav1.8 activity attenuates signs of neuropathic pain including mechanical hypersensitivity (Lai et al., 2004). Interestingly, Nav1.8 mRNA expression is decreased in nociceptors after nerve injury (Okuse et al., 1997). How can these disparate findings be reconciled? One possible explanation is that the regulation of Nav1.8 mRNA transcription and translation are decoupled and while transcription is decreased after nerve injury, translation efficiency is enhanced. Evidence for this idea comes from studies demonstrating that Nav1.8 mRNA is transported into axons after injury where it can be locally translated at sites of injury (Thakor et al., 2009; Ruangsri et al., 2011; Hirai et al., 2017). This provides an explanation for why what could be interpreted as a loss of function phenotype (decreased mRNA expression) in the cell body may result in a gain of function phenotype at the site of injury (increased local translation of Nav1.8) leading to ectopic generation of action potentials and enhanced excitability to mechanical stimulation. This shift in the site of functional expression of Nav1.8 after injury may depend on changes in 3′ untranslated region (UTR) splicing that generate a longer 3′UTR after injury that is more readily targeted to the axonal compartment (Hirai et al., 2017).

The example of translation regulation of Nav1.8 expression in neuropathic pain is just one set of studies among a growing body of literature that highlights how translation regulation signaling controls nociceptor sensitization (Khoutorsky and Price, 2017) as well as neuronal plasticity along the pain pathway in the spinal cord and brain (Price and Inyang, 2015). The first translation regulation signaling pathway that was extensively studied in the context of nociceptor plasticity was the mechanistic target of rapamycin complex 1 (mTORC1, (Price et al., 2007; Jimenez-Diaz et al., 2008; Geranton et al., 2009; Melemedjian et al., 2010)) which is composed of the mTOR kinase and a set of scaffolding proteins that includes raptor. The specific mTORC1 inhibitor rapamycin inhibits nociceptor plasticity induced by a number of pain promoting endogenous molecules (e.g. nerve growth factor (NGF) and interleukin 6 (IL6)) and mTORC1 activity is increased in the dorsal root ganglion (DRG) in response to nerve injury (Melemedjian et al., 2010; Melemedjian et al., 2011). Accordingly, inhibition of mTORC1 with rapamycin analogues (called rapalogues) also reduces neuropathic pain (Jimenez-Diaz et al., 2008; Geranton et al., 2009). Unfortunately, as will be discussed in more detail below, long-term inhibition of mTORC1 activity leads to robust feedback activation of the mitogen activated protein kinase (MAPK) signaling pathway which is also a well-known promoter of nociceptor excitability (Melemedjian et al., 2013). Hence, manipulating this specific target is fraught with issues that make it unlikely that direct or allosteric mTORC1 inhibitors will ever be used for pain. However, the discovery of mTORC1 as a key regulator of nociceptive plasticity has led to a blossoming of other studies that highlight additional targets that have the potential to generate new pain therapeutics.

The basic neurobiology of translation regulation in chronic pain has been reviewed recently (Khoutorsky and Price, 2017) and will not be comprehensively reviewed again here. Our purpose in the remainder of this review will be to focus on: 1) why translation regulation signaling pathways are excellent targets for therapeutics in general and why they are exciting for chronic pain, 2) how gaining a better insight into mRNAs that are translated during chronic pain conditions in nociceptors is likely to reveal additional high-quality targets and 3) to highlight specific pharmacological targets in the translation regulation pathway and critically review their potential to yield new therapeutics.

## The emerging evidence of specificity in translation regulation signaling – Why are translation regulation signaling factors excellent pharmacological targets

A widely held view of translation regulation in cells is that mRNAs are transcribed in the nucleus and exported to the rough endoplasmic reticulum (rER) or to sites of free ribosomes in the cytosol and proteins are then generated from these mRNA through a process where protein production rate reflects the abundance of available transcript. While this may be the case for some mRNAs, it is now understood that translation depends on spatial and temporal factors that have very specific effects, in many case, on small subsets of mRNAs (Sonenberg and Hinnebusch, 2009). Some of this specificity emerges from mRNA localization where specific sequences in the mRNA, usually in the 3′UTR, promote mRNAs association with mRNA-binding proteins that then transport these mRNAs to distal locations in cells. In neurons, these targeting factors are known to localize some, but certainly not all, mRNAs to dendrites or axons where they can then be translated on demand to regulate neuronal plasticity (Costa and Willis, 2017; Van Driesche and Martin, 2018).

Additional specificity emerges from temporal control of translation in cells. Temporal control of translation in neurons emerges mostly from activation of neurotransmitter and/or chemokine/cytokine/growth factor receptors that then modulate the activity of intracellular kinases that regulate translation through mechanisms that will be described in detail below. These kinases phosphorylate proteins that bind to mRNAs either on the 5′ or 3′ end. On the 5′ end these proteins include eukaryotic initiation factors (eIFs) such as eIF4E, eIF4G and eIF4A, which collectively form a complex that is referred to as the eIF4F complex. These proteins also include the 4E binding proteins (4EBPs) which tonically suppress the formation of the eIF4F complex until they are phosphorylated by mTOR (Khoutorsky and Price, 2017). On the 3′ end are proteins that regulate the length of the poly-A tail. The best characterized proteins in this family are the poly-A binding proteins (PABPs, (Gray et al., 2015)) and the cytoplasmic polyadenylation binding proteins (CPEBs, (Richter, 2007)). While specific phosphorylation events that regulate poly-A tail length have not been characterized in detail, it is known that kinases such as calcium/calmodulin activated protein kinases (CaMKs) are able to regulate poly-A tail length in neuronal plasticity via a mechanism that involves CPEBs and PABPs (Atkins et al., 2005; Kaczmarczyk et al., 2016).

From the perspective of developing therapeutics, these intricate signaling pathways that regulate translation provide many opportunities for intervention (for a recent comprehensive review on the topic see: (Bhat et al., 2015)). Many distinct kinases regulate and/or directly phosphorylate proteins that compose or inhibit the eIF4F complex. Additionally, some of the proteins that bind to mRNAs are enzymes (e.g. eIF4A, which is an RNA helicase) that can be targeted by specific inhibitors (Parsyan et al., 2011). More importantly, it is now becoming clear that each of these individual phosphorylation events leads to specific translation regulation of a subset of mRNAs. Perhaps the best example of this is MAPK interacting kinase (Mnk1/2, encoded by the *MKNK1* and *MKNK2* genes, respectively) mediated phosphorylation of eIF4E (Wang et al., 1998; Pyronnet et al., 1999; Waskiewicz et al., 1999). Mnk1 and 2 phosphorylate eIF4E at serine 209. No other known kinase phosphorylates this site and this is the only phosphorylation site on eIF4E. Inhibition of eIF4E phosphorylation by Mnk1/2 with pharmacology or genetics does not change bulk translation in cells but it does suppress the translation of a small subset of mRNAs that are critically involved in carcinogenesis, immune responses and intrinsic neuronal plasticity, depending on the cell type (Furic et al., 2010; Herdy et al., 2012; Gkogkas et al., 2014; Moy et al., 2017). Therefore, targeting eIF4E with inhibitors of Mnks is unlikely to have strong effects on most cells (eIF4E phosphorylation null and Mnk1/2 double knockout mice are viable and show no developmental deficits) in the body, but it does alter the response to situations where cellular plasticity is induced (Furic et al., 2010; Herdy et al., 2012; Gkogkas et al., 2014; Moy et al., 2017). This example demonstrates that targeting specific translation regulation signaling events is able to interfere with shifts in translation efficiency for subsets of mRNAs that are involved in distinct cellular responses. Gaining further insight into which signaling pathways are involved in particular types of phenotypic plasticity and whether these signaling events are required for the initiation or persistence of these cellular changes will be paramount to developing drugs that target translation regulation.

## New technologies to detect mRNAs that are translated in specific conditions on a genome wide scale

Compelling evidence indicates that transcriptional and translational regulation are decoupled in many cellular contexts and that mRNA levels do not accurately reflect protein abundance due to differential translational efficiencies and protein half-lives (Schwanhausser et al., 2011; Edfors et al., 2016; Liu et al., 2016; Fortelny et al., 2017). This increases the need for new techniques/technologies to identify translating mRNAs in specific cells *in vitro* and *in vivo*. Ingolia et al., have developed a new approach, named ribosome footprinting (RFP) based on the deep sequencing of ribosome-protected mRNA fragments that allows accurate monitoring of translation *in vitro* (Ingolia et al., 2009). Unlike polysome profilling, which is only based on the separation of translated mRNAs according to the number of bound ribosomes, RFP provides information about the precise positions of ribosomes on specific transcripts facilitating discovery of alternative start codons, upstream Open Reading Frame (uORF) and/or translational readthrough of the canonical stop codon (Ingolia et al., 2012). Moreover, each footprint generated by a single ribosome indicates which mRNA was being translated, allowing an accurate quantification of the translation efficiencies for each mRNA on a transcriptome wide scale. Although the RFP technology constitutes a dramatic advance in the understanding of translation regulation at a single codon resolution, it does not allow the detection of translating mRNAs in a cell type-specific manner. It is clear that the CNS and PNS are composed of hundreds, if not thousands, of cell types yet neurological disorders can be caused by “genomic” dys-regulation in only a single cell type. How can you translationally profile this single cell type among a complex tangle of cell types in the nervous system? To overcome this problem a new technology named Translating Ribosome Affinity Purification (TRAP) methodology enables the identification of translated mRNAs in a cell type of interest. This methodology involves the expression of an enhanced green florescent protein (eGFP) –L10a ribosomal transgene, which allows tagging of polysomes and subsequent immunoaffinity purification of ribosome bound mRNAs (Heiman et al., 2014). The first transgenic lines were created using bacterial artificial chromosome (BAC) enabling translational profiling of multiple cell-types in the CNS (http://www.gensat.org/TRAP_listing.jsp) Recently, a Cre-inducible TRAP line was characterized (Zhou et al., 2013), creating the possibility to identify translated mRNAs in any given cell-type. Until now, TRAP technology was mostly used for a comprehensive profiling of the mRNA translation landscape in the targeted cell type (Doyle et al., 2008; Morel et al., 2017; Ouwenga et al., 2017; Sakers et al., 2017). However, this technology could also help to gain a better understanding on how changes in the translatome in specific cell types can lead to the development of neurological disorders (Thomson et al., 2017). Finally, we could also imagine that mRNAs showing high translation efficiencies under specific conditions would represent potentially new targets for the development of new therapeutics (Thomson et al., 2017). We anticipate that the use of these technologies in the neurobiology of pain area will revolutionize our understanding of the cellular basis of pain plasticity.

## The potential of specific translation signaling mechanisms as therapeutic targets for pain

The following paragraphs will discuss specific targets for pharmacological manipulation of translation that have potential as pain therapeutics. These targets are summarized in Table 1.

### mTORC1 and mTOR kinase inhibitors

**Fig. 2:**
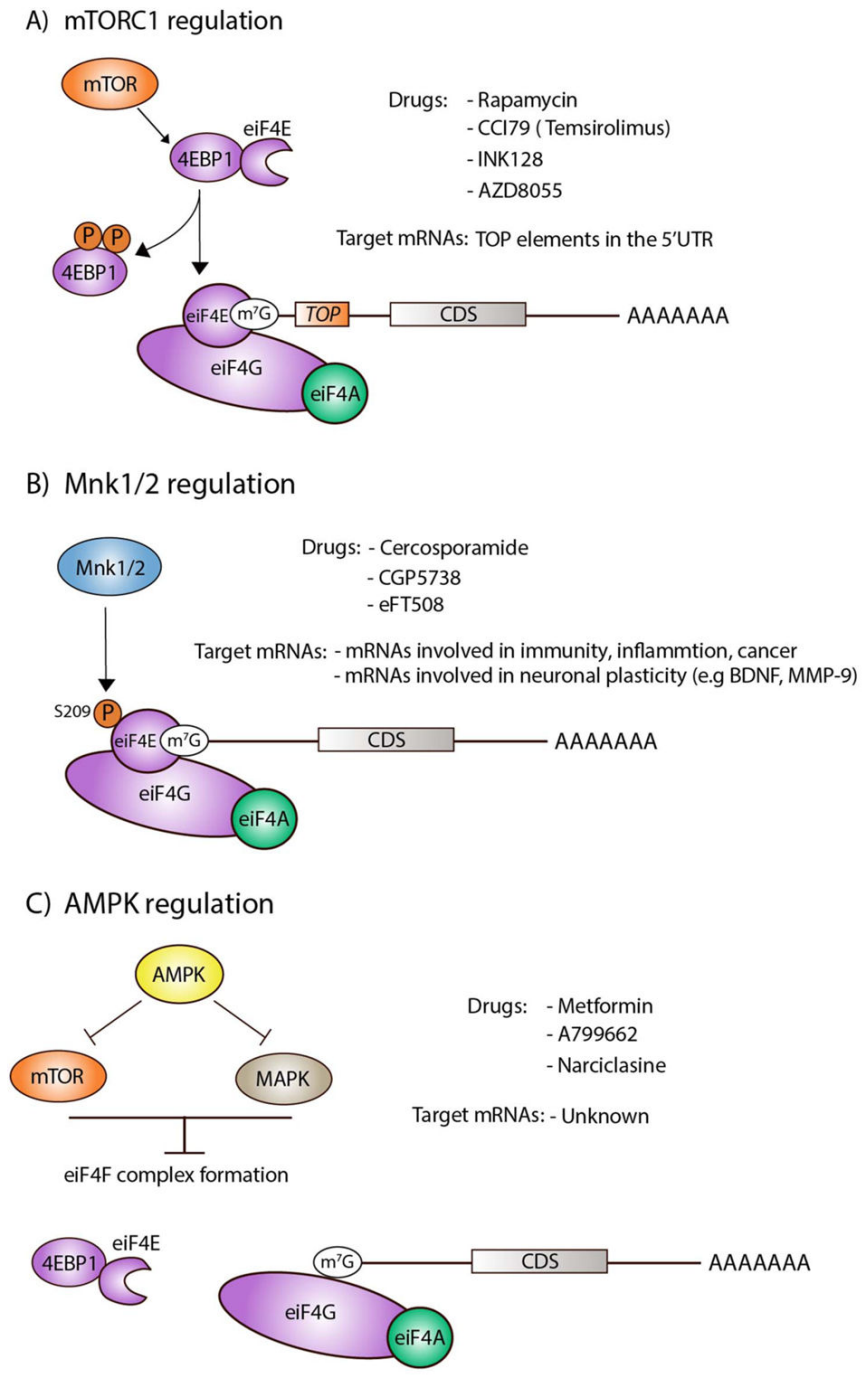
Targeting strategies for mTORC1, Mnk1/2 and AMPK Summary diagram showing (A) mTORC1 regulation and its primary target mRNAs, (B) Mnk1/2 regulation and its primary target mRNAs and (C) AMPK mediated inhibition of mTOR and MAPK signaling.

mTOR is a master regulator of protein synthesis integrating a variety of environmental cues to regulate cellular homeostasis. mTOR forms at least two multiprotein complexes known as mTOR complex 1 (mTORC1) and mTOR complex 2 (mTORC2). mTORC1 is well understood and recognized as an environmental sensor with sensitivity to rapamycin (Fig. 2A). This first generation of mTOR inhibitors present the same binding sites for mTOR and FKBP12 and are then called rapalogs (i.e., rapamycin and its analogs). Rapalogs include CCI-779 (temsirolimus), and RAD-001 (everolimus). Rapamycin has shown efficacy in various models of inflammatory and neuropathic pain (Obara et al., 2011; Xu et al., 2011) many of which are also associated with mTOR dysregulation (Price et al., 2007; Jimenez-Diaz et al., 2008; Geranton et al., 2009; Melemedjian et al., 2011; Khoutorsky and Price, 2017). The utilization of other rapalogues, such as CCI-779, also acutely relieves mechanical pain (Obara et al., 2011). However, long-term treatment with rapalogues can cause mechanical hypersensitivity and pain via a feedback activation of the ERK kinase (Melemedjian et al., 2013), which is a well-known mediator of nociceptor hyperexcitability (Ji et al., 2009).

These limitations of the allosteric inhibitors of mTORC1, which were long-ago noted in the cancer field (Carracedo et al., 2008a), motivated the development of second generation mTOR inhibitors that target the kinase domain, blocking both mTORC1 and mTORC2 activity (Benjamin et al., 2011). Unlike rapamycin, these ATP-competitive inhibitors target the catalytic site of the enzyme, promoting a more potent and sustained inhibition of mTORC1 and mTORC2. Such second-generation inhibitors include INK128 and AZD8055 whose efficacy is currently being evaluated in patients for the treatment of various cancers. The first studies suggested that feedback-dependent activation of MAPK was fully abrogated in AZD8055-treated cells (Renshaw et al., 2013). However, recent findings suggest that long-term treatment with AZD8055 also leads to a feedback activation *via* receptor-tyrosine kinases such as EGFR (Wei et al., 2015) which has been recently shown to play a crucial in nociceptive plasticity in rodents, humans and flies (Martin et al., 2017). For these reasons, our opinion is that allosteric or direct inhibitors of mTOR will likely have little utility for the treatment of pain in the clinic despite a clear case for mTOR as a mechanistic link between injury and nociceptive plasticity.

### Dual MAPK/mTOR inhibitors

Another therapeutic strategy consists of dual inhibition of the MAPK and/or PI3K and mTOR pathway. PI3K and mTOR belong to the PI3K-related kinases (PIKK) superfamily and share structural domains which facilitates the development of drugs targeting both kinases. The dual PI3K-mTOR inhibitors target the p110 subunit of PI3K as well as mTOR inhibiting the pathway both upstream and downstream of AKT. This strategy prevents an over-activation of AKT following the inhibition of the mTORC1–S6K–IRS1 negative feedback loop previously described with rapalogues (Carracedo et al., 2008a; Melemedjian et al., 2013). However, the potential toxicities associated with these dual inhibitors presents a limitation given the diverse functions PI3Ks isoforms. Consequently, it is generally accepted that inhibitors with more selectivity for mTOR would have a better tolerability profile than the dual inhibitors (Pallet and Legendre, 2013).

A second approach is to create therapeutics that are composed of two molecules for simultaneous targeting of MAPK and PI3K/mTOR signaling. This approach was pioneered to overcome, again, limitations related to feedback signaling when only one arm of these convergent signaling pathways was inhibited (Carracedo et al., 2008a; Carracedo et al., 2008b). While there have been some successes in the use of this approach in the oncology space (Kinkade et al., 2008; Shimizu et al., 2012), toxicities are clearly a major issue in the clinic (Mita et al., 2017). While this approach holds promise, it remains to be seen if drug combinations or innovative ways of creating bivalent compound mixtures (Galban et al., 2017) will be able to create therapeutics that have a safety profile that will allow use in pain patients.

### Mnk-eIF4E

Mnk1 and 2 phosphorylate eIF4E at serine 209 and inhibition of eIF4E phosphorylation by Mnk1/2 using pharmacological or genetic tools can alter translation efficiencies of mRNAs involved in neuronal plasticity (Gkogkas et al., 2014; Moy et al., 2017) (Fig. 2B). We have recently demonstrated that mice lacking the phosphorylation site for Mnk1/2 on eIF4E show a strong deficit in pain hypersensitivity in inflammatory pain models, an effect that is recapitulated by Mnk1/2 double knockout mice (Moy et al., 2017). This effect can also be produced with pharmacological blockade of Mnk signaling. Cercosporamide is an antifungal agent that was identified during a chemical screen for its ability to selectively inhibit Mnk1/2 (Konicek et al., 2011). Our study showed that NGF- and IL-6-induced increases in neuronal excitability *in vitro* and mechanical sensitivity *in vivo* were attenuated after treatment with the Mnk1/2 inhibitor, cercosporamide (Moy et al., 2017) an effect that may be related to a decrease in BDNF translation (Moy et al., 2018). In spite of its efficacy in inhibiting eIF4E phosphorylation, the poor bioavailability and lack of specificity of cercosporamide motivated the development of more potent and specific Mnk1/2 inhibitors with drug like-properties. eFT508 is a new generation Mnk1/2 inhibitor with a low nM potency and a good specificity profile, developed by eFFECTOR (Dreas et al., 2017). The compound is in clinical development where it has shown good tolerability in a dose-escalation study in patients and is currently being tested for the treatment of colorectal cancer in Phase II (Thompson et al., 2017). Importantly, eFT508 shows high potency at Mnk1/2 and achieves full inhibition of eIF4E phosphorylation *in vivo* at doses less than 10 mg/kg (Webster et al., 2015). As opposed to mTOR inhibitors, long-term Mnk1/2 inhibition does not seem to induce feedback activation of the mTOR pathway and eFT508 is able to block the over-activation of ERK induced by rapalogue treatment in cancer cells lines (Webster et al., 2015). While this compound has not been tested in pain models, the combination of existing genetic and pharmacological evidence makes this signaling pathway an exciting new target. An important aspect of Mnk1/2 targeting is that this pathway is unlikely to have an influence on bulk translation and apparently only influences a small subset of mRNAs that are involved in neuronal plasticity, immunity and oncogenesis (Furic et al., 2010; Herdy et al., 2012; Gkogkas et al., 2014; Moy et al., 2017). Therefore, targeting eIF4E phosphorylation with pharmacological inhibitors of Mnks could represent a future therapeutic avenue for chronic pain treatment.

### AMPK

The adenosine monophosphate activated protein kinase (AMPK) is a master regulator of cell metabolism that is a heterotrimeric kinase which negatively regulates MAPK and PI3K/mTOR signaling and modulates lipid metabolism in all cells (Hardie et al., 2012) (Fig. 2C). AMPK can be activated by indirect stimulators that act primarily through mitochondrial actions to alter cellular AMP/ATP levels (e.g. metformin) or by direct allosteric modulators that increase kinase activity via a variety of mechanisms (Xiao et al., 2013). This latter group of compounds is a very active area of discovery, mostly in the metabolism and cancer spaces (Cameron and Kurumbail, 2016). Merck recently developed a very specific and potent activator of AMPK, MK-8722, which has favorable effects on metabolism and few side-effects with 6 months of dosing in rodents or non-human primates. The compound did produce a benign cardiac hypertrophy consistent with what is frequently seen in elite athletes that was attributed to on-target effects (Myers et al., 2017).

A large number of pharmacological and genetic studies have now been completed in the pain space indicating that AMPK activators might be used for the treatment of neuropathic or other persistent pain states (Price and Dussor, 2013; Price et al., 2015; Asiedu et al., 2016). In the peripheral nervous system, AMPK activation strongly inhibits both mTOR and MAPK signaling (Melemedjian et al., 2011; Tillu et al., 2012), reduces nociceptor excitability (Melemedjian et al., 2011; Tillu et al., 2012; Asiedu et al., 2017; Burton et al., 2017) and reverses or inhibits the pain promoting effects of nerve injury or inflammation (Melemedjian et al., 2011; Tillu et al., 2012; Russe et al., 2013; Bullon et al., 2015; Ma et al., 2015; Maixner et al., 2015; Song et al., 2015; Burton et al., 2017). Importantly, studies using specific genetic ablation of α subunits of the heterotrimeric kinase demonstrate that reducing AMPK activity enhances inflammatory pain and that peripherally expressed AMPK is needed for the beneficial effects of AMPK activators (Russe et al., 2013). AMPK activation in the CNS has also been linked to positive outcomes in chronic pain models with some of the most compelling evidence linking AMPK activation to enhance glutamate clearance via an astrocyte-mediated mechanism (Maixner et al., 2015). Finally, a very recent study has linked AMPK activation to the beneficial effects of exercise on chronic pain (King-Himmelreich et al., 2017). Collectively these studies point to a very positive outlook for the potential of developing potent and specific AMPK activators for the treatment of pain.

### PABP

The targets mentioned above primarily function through regulation of the 5′ end of the mRNA via an action on the eIF4F complex. Regulation of translation also occurs at the 3′ end with one of the best studied mechanisms being regulation of the poly-A tail. This regulation is largely governed by two types of proteins, the poly-A binding proteins, PABPs, and the cytoplasmic polyadenylation element binding proteins, CPEBs. CPEBs have been the focus of intense investigation in the neuronal plasticity due to their effect on activity-dependent poly-A tail lengthening, an effect which should enhance the translation efficiency of targeted mRNAs. Several studies, including studies examining nociceptor plasticity, have linked CPEB activity to neuronal plasticity and enhancement of pain responses (Wu et al., 1998; Si et al., 2003b; Si et al., 2003a; Theis et al., 2003; Bogen et al., 2012; Ferrari et al., 2012; Ferrari et al., 2013). From a pharmacological perspective, targeting CPEBs is challenging because upstream kinases that regulate CPEB activity have not been identified with great specificity. Having said that, CaMKIIα phosphorylates CPEB (Kaczmarczyk et al., 2016) and CPEB activity regulates CaMKIIα translation (Wu et al., 1998) making this kinase an interesting potential target for regulation of CPEB activity and CPEB-mediated neuronal plasticity, including pain plasticity (Bogen et al., 2012; Ferrari et al., 2012; Ferrari et al., 2013).

**Fig. 3:**
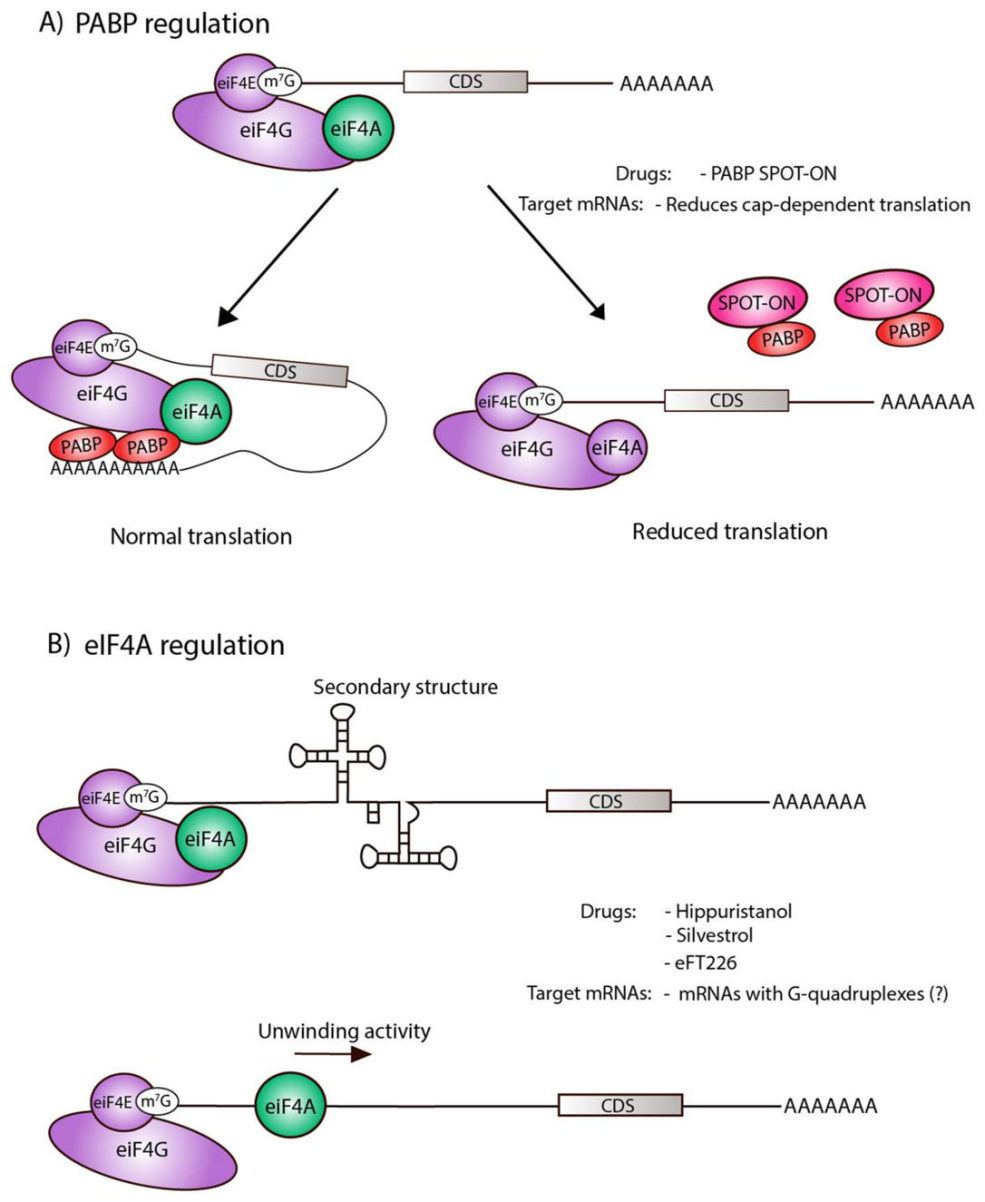
Targeting strategies for PABP and eIF4A Summary diagram showing targeting strategies for PABP (A) which is involved in regulation of poly-A tail length and mRNA circularization and (B) eIF4A which is an RNA helicase putatively involved in unwinding 5′ UTR G-quadruples structures.

PABP proteins bind to the poly-A tail and regulate its length. These proteins also play important roles in RNA localization and translation efficiency (Gray et al., 2015). This family of proteins have not traditionally been thought of as druggable targets but a recent study demonstrates a new technology that may open new vistas in the pharmacological targeting of RNA binding proteins in general and PABPs in particular (Fig. 3A). This work demonstrated that a stabilized RNA molecule (called a SPOT-ON) that specifically targets the PABP high affinity RNA binding site is capable of disrupting PABP-RNA interactions and decreases translation efficiency in cells, including mouse DRG neurons (Barragan-Iglesias et al., 2018). When this stabilized RNA molecule was tested *in vivo* it profoundly reduced mechanical hypersensitivity induced by capsaicin, nerve growth factor (NGF) and hindpaw incision (Barragan-Iglesias et al., 2018). Clearly more work is needed to develop this novel area of pharmacology but the potential here is very high because this pathway, perhaps surprisingly, also appears to target only a select pool of mRNAs that are involved in plasticity and due to the strong efficacy of the PABP-interfering RNA mimetic in mouse pain models (Barragan-Iglesias et al., 2018).

### eIF4A

The final member of the eIF4F complex discussed here is the RNA helicase, eIF4A. This enzyme plays a key role in translation by unwinding secondary structure in 5′ UTRs and potentially other regions of the mRNA (Parsyan et al., 2011). It is controversial whether eIF4A has specific effects on certain structures or more generally influences bulk translation in cells (Rubio et al., 2014). A recent study identified G-quadruplex structures in 5′ UTRs as a key target for eIF4A activity as it pertains to translation efficiency (Wolfe et al., 2014) but other studies have suggested other functions for eIF4A (Sokabe and Fraser, 2017). Nevertheless, a variety of eIF4A inhibitors have been developed and these compounds have strong effects on translation in cells with very potent activities suggesting a high degree of specificity ((Parsyan et al., 2011), Fig. 3B). To date, these compounds have been used sparingly in *in vivo* models owing largely to their limited bioavailability. There are several eIF4A isoforms in mammalian genomes (*EIF4A1, EIF4A2* and *EIF4A3* in humans) and emerging evidence suggests that these might be targetable individually. In this regard, eFFECTOR has also developed specific eIF4A1 inhibitors that may have utility as regulators of nociceptor plasticity (Ernst et al., 2017). This hypothesis remains to be tested.

### eIF2α – integrated stress response

**Fig. 4:**
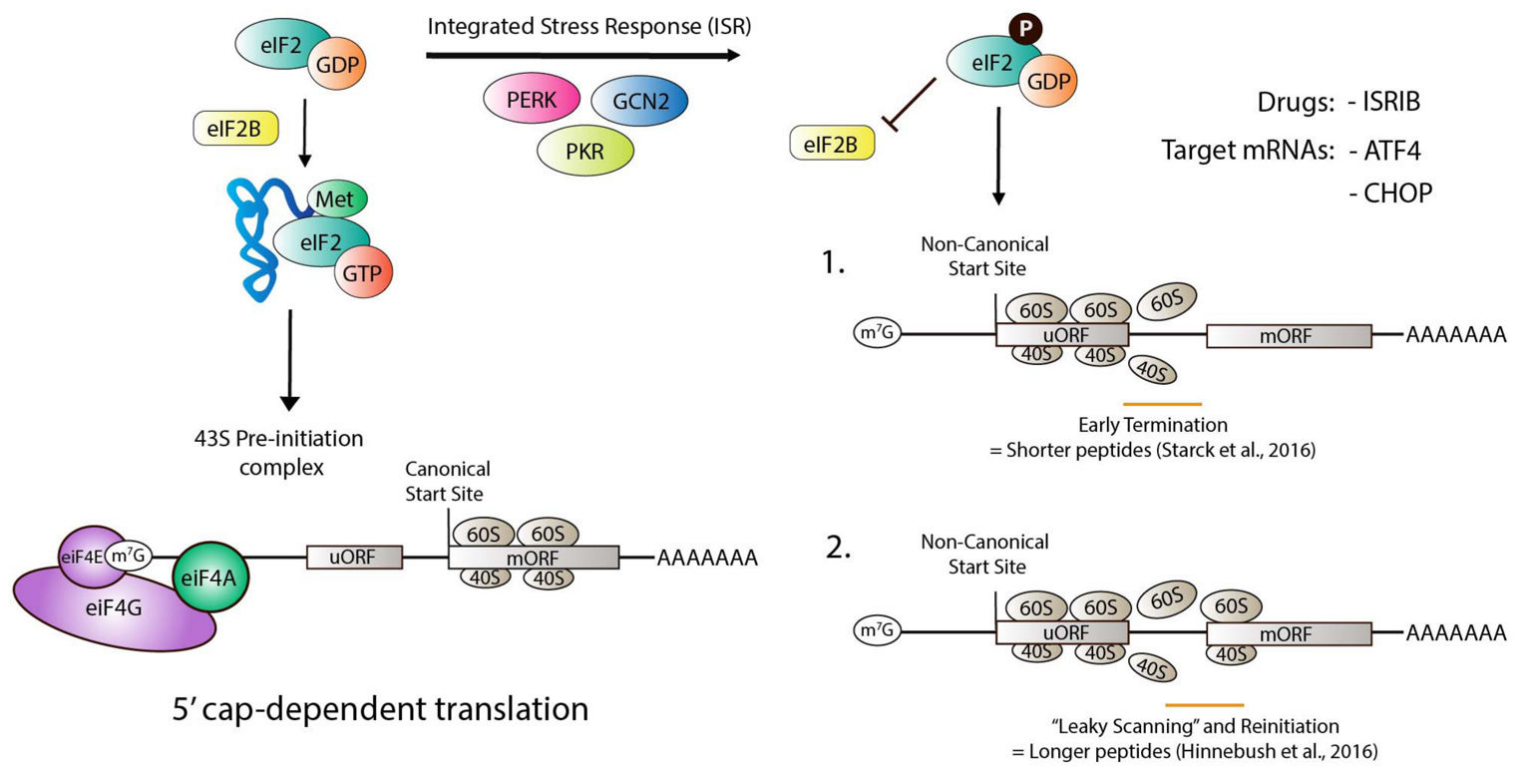
Targeting strategies for eIF2α/Integrated Stress Response Summary diagram showing how eIF2α phosphorylation leads to disruption of cap-dependent translation and engagement of translation via non-canonical start sites in the 5′ UTR of mRNAs.

eIF2α is a negative regulator of cap-dependent translation when it is phosphorylated by upstream kinases (Wek et al., 2006) that are now widely recognized as key regulators of the integrated stress response (ISR, Fig. 4, (Pakos-Zebrucka et al., 2016)). In its unphosphorylated state, eIF2α promotes translation initiation at canonical open reading frames (ORFs) but when the protein is phosphorylated it suppresses this form of translation and instead promotes translation through non-canonical ORFs, which can include alternative start site (Costa-Mattioli et al., 2005; Costa-Mattioli et al., 2007) or upstream ORFs (uORFs, (Starck et al., 2016)). Hence, eIF2α promotes a shift in translation efficiency from cap-dependent protein synthesis to utilization of alternative start codons which lead to the production of either short peptides derived from uORFs or longer proteins that have different amino acid sequences at their N-terminals due to origination of translation at an alternative start codon, usually upstream of the canonical start codon (Fig. 4). Since eIF2α phosphorylation is induced by the ISR, this cellular pathway appears to play an important role in producing protein diversity in cells, an area of biology that is rapidly evolving and has been greatly facilitated by the advent of the RFP technology (Starck et al., 2016).

A role for eIF2α in pain has emerged from 2 lines of investigation. First, the use of a mouse that is hypomorphic for eIF2α phosphorylation has revealed an important role of eIF2α phosphorylation in thermal nociception and in inflammation induced pain plasticity (Khoutorsky et al., 2016). Second, investigations in models of diabetic neuropathic pain have revealed a very robust activation of eIF2α phosphorylation in DRG axons suggesting a functional role of eIF2α in diabetic neuropathic pain (Inceoglu et al., 2015). Indeed, molecular chaperones that interfere with the cellular consequences of ISR induction have a beneficial effect in diabetic neuropathic pain models (Inceoglu et al., 2015). Interestingly, a specific inhibitor of the ISR, ISRIB, has been developed and widely used in other models of cellular and neuronal plasticity (Sidrauski et al., 2015) and might be a promising therapeutic avenue for many types of chronic pain given the emerging evidence of eIF2α involvement in inflammatory and neuropathic pain.

### Ragulator – vacuolar ATPase

mTOR forms a super-complex of proteins on the lysosomal membrane and its transactivation depends on the interaction between multiple factors *via* different pathways. Sabatini and colleagues showed that GTPases and GEFs (Guanine Nucleotide Exchange Factor) play a critical role in mTOR activation in response to specific environmental conditions such as nutrient availability, cellular stressors and growth factors(Wolfson and Sabatini, 2017). These GTPases and GEFs include the Rag family of GTPases and the mTOR associated protein Rheb. A previous study showed that Rheb expression is transiently increased in a model of peripheral inflammation which correlated with an increased mTOR activity (Norsted Gregory et al., 2010). Indeed, over-activation of AKT leads to a disinhibition of Rheb which in turn binds and sequesters the endogenous inhibitor of mTOR, FKBP-38 (Bai et al., 2007). Other mTOR associated GTP binding proteins are emerging as potential regulatory targets for mTOR signaling. Specifically, since the discovery of the Rag GTPases as components of the mTORC1 pathway, a large number of proteins have been identified as playing a role upstream of the mTORC1 complex. The Ragulator is a pentameric complex composed of p18, p14, HBXIP, C7orf59, and MP1 (Lamtor1-5) which controls the lysosomal localization and nucleotide loading state of the Rag GTPases (Sancak et al., 2010; Bar-Peled et al., 2012). Ragulator interacts with the vacuolar H^+^ adenosine triphosphate ATPase (v-ATPase), which acts as a positive regulator of the pathway through an unknown mechanism (Zoncu et al., 2011) Intriguingly, a recent finding demonstrates that inhibiting the proton pump v-ATPase with bafilomycin A1 reduces bone pain induced by multiple myeloma (Hiasa et al., 2017) although the authors of this study did not attribute these effects of bafilomycin A1 to mTOR. Although the role of the Rag-Ragulator complex in mTORC1 activation has been clearly demonstrated in other systems, the exact role of this pathway in chronic pain remains to be elucidated, but is pharmacologically tractable as evidenced with bafilomycin A1. Since this signaling complex lays upstream of mTORC1 activation, it may be capable of disrupting altered mTORC1 signaling in pathological neuronal plasticity without inducing feedback signaling mechanisms that exacerbate this plasticity.

## Concluding remarks

The studies described above demonstrate a robust pipeline of pharmacologically tractable targets in the translation control pathway for the potential generation of pain therapeutics. Given the rapidly growing literature on the role of translation regulation in pain plasticity in general, and in nociceptor sensitization specifically, we propose that this area is ripe for interventional manipulation and potential disease modification for pain. As technologies that allow for translational profiling of these cells emerge and are utilized, we also anticipate that this will create even more abundant potential intervention points for disruption of plasticity that maintains chronic pain states.

## Figures and Tables

**Table 1:** List of mechanistic targets for inhibition of specific translation regulation signaling pathways and drugs that target those mechanisms. Advantages and disadvantages of each of those signaling mechanisms are also considered in the context of pain and/or other known side effects.

Target	Drug(s)	Advantages	Disadvantages
mTORC1	Rapamycin Everolimus Rapalogues	Very specific for mTORC1 Decreases nociceptor excitability in the short term Alleviates pain with acute treatment in many preclinical models	Strong immune suppressant Causes feedback activation of ERK signaling Losses effect on nociceptor excitability with repeated dosing likely due to feedback signaling Some evidence of causing a CRPS-like syndrome in some patients
mTOR kinase inhibitors	Torin 1 INK128, AZD8055 AZD2014	Inhibits both mTORC1 and mTORC2 May cause less feedback signaling than rapalogues due to engagement of mTORC2	Largely untested in preclinical pain models Clinical findings suggest that feedback signaling activation is still a significant problem
Dual MAPK/mTOR inhibitors	Multi drug combinations including a mTOR or PI3K inhibitor and a MAPK inhibitor	Theoretically prevent feedback signaling by simultaneously blocking both pathways Enhanced efficacy versus mTOR inhibitors	Pharmacokinetic matching of 2 drugs is challenging Strongly inhibit cap-dependent translation by blocking multiple pathways Severe side-effect profiles based on cancer clinical trials.
Mnk-eIF4E	Cercosporamide eFT508	Very specific effect on eIF4E phosphorylation Only inhibits translation of a select subset of mRNAs that seem to be involved in plasticity and/or inflammation Strong inhibition of nociceptor sensitization eFT508 is in Phase II clinical trials for cancer	Side effect profile is not well understood, but transgenic mice lacking this pathway are viable, develop normally and have few deficits in synaptic physiology Relatively small number of molecules that inhibit Mnk have been developed, more SAR needed
AMPK	Metformin AICAR A769662 ZLN-024 MK-8722	Good safety profile Inhibits nociceptor excitability Reverse established chronic pain in multiple preclinical models Early treatment with AMPK activators prevents development of chronic pain and late treatment in neuropathic models has disease modifying properties May have positive effects on metabolism that are also advantageous in the context of chronic pain	AMPK acts on many pathways so not clear if effect is due to translation signaling Known cardiac hypertrophy effect with chronic dosing that resembles effects seen in elite athletes
PABP	PABP SPOT-ON Cordycepin	PABP SPOT-ON is an RNA mimetic with presumed high specificity for inhibition of PABP interaction with mRNAs *In vivo* effect of blocking PABP function is inhibition of injury-induced pain amplification	Still very early in development phase but technology can be used to target a broad variety of RNA binding protein / mRNA interactions
eIF4A	Panteamine A Hippuristanol Silvestrol eFT226	eIF4A inhibitors block helicase activity and may be specific for a subset of mRNAs that require eIF4A for efficient translation Compounds that are specific for individual eIF4As, such as eFT226 which is specific for eIF4A1, can be developed	Largely untested in the context of pain General eIF4A inhibitors strongly attenuate translation in cells suggesting possibility of severe side effects for some compounds.
eIF2α	BTdCPU (stimulates eIF2α phosphorylation) ISRIB (inhibits the ISR)	eIF2α phosphorylation is induced in sensory nerves in diabetic models and blocking this pathway alleviates diabetic neuropathic pain eIF2α phosphorylation is a hallmark of induction of the integrated stress response (ISR) and promotes translation of mRNAs through non-canonical start sites leading to the generation of novel peptides eIF2α seems to regulate functional expression of TRPV1 and regulates heat sensitivity of nociceptors	eIF2α modulators have not been widely used in preclinical pain models so effects are largely unknown Compounds are available to stimulate eIF2α phosphorylation (BTdCPU) or mitigate the effects of eIF2α phosphorylation (ISRIB) so there is great potential to explore this pathway in more detail.
Ragulator/vacuolar ATPase	Bafilomycin A1	Bafilomycin A1 is an antibiotic that has anti- hyperalgesic effects in bone cancer models that have been attributed to ASICS function Inhibiting mTOR upstream of kinase activity by regulating the ragulator complex may modulate mTOR activity without engaging feedback signaling seen with other mTOR inhibiting strategies Other opportunities to interfere with ragulator GTPases using small molecules will likely emerge	Many of the ragulator complex proteins have only recently been discovered so relatively little is known about possibilities for pharmacology at these targets Compounds that inhibit the function of the ragulator complex may also interfere with lysosomal function since this complex sits on the lysosomal membrane
Cap-dependent translation inhibitors	4EGI-1 ribavirin	Interfere with eIF4F complex binding to the cap of mRNAs to inhibit most cap-dependent translation 4EGI-1 is effective with local injection (peripheral or spinal) in many pain models	Toxicity with systemic dosing likely to be high with chronic use although ribavirin is used clinically for viral infections (ribavirin mimics the 5′ cap structure to interfere with eIF4F function)
